# Supplementary material for: Functional Comparison between Endogenous and Synthetic Notch Systems
Source: ACS Synth Biol. 2022 Sep 15;11(10):3343–53. doi: 10.1021/acssynbio.2c00247 (PMC9594772; doi:10.1021/acssynbio.2c00247)
Supplement: Supplementary file 1 — sb2c00247_si_001.pdf [file sb2c00247_si_001.pdf]

# Supporting Information

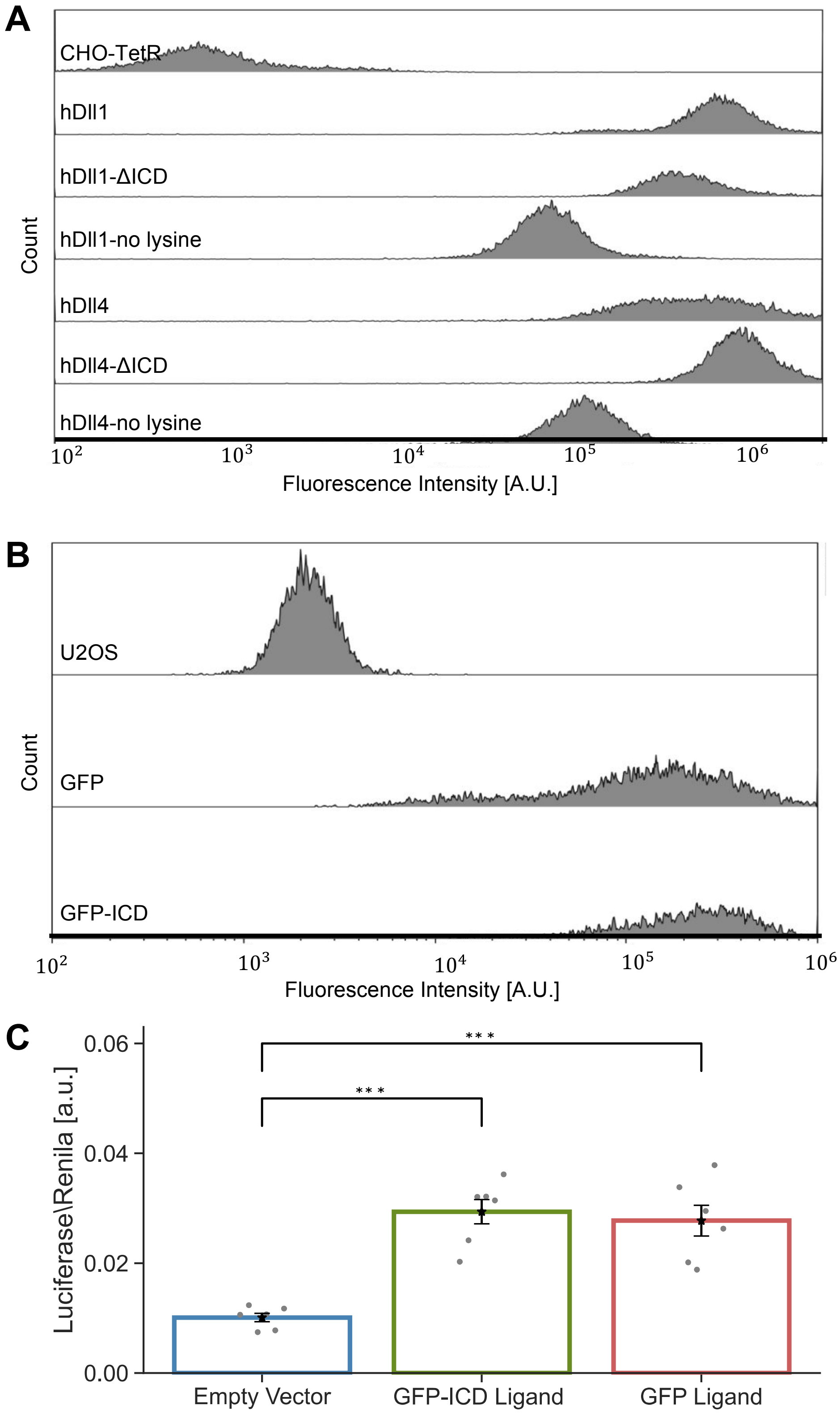

**Figure S1. FACS analysis of fluorescently tagged ligands. (A)** Comparison of the fluorescence of CHO-TetR cells expressing different ligand variants tagged with mCherry (as indicated for each row). Control cells (CHO-TetR) that do not express fluorescent protein are shown in the top row. **(B)** Comparison of the fluorescence of U2OS cells expressing either the GFP or GFP-ICD ligand of the aa-synNotch system (as indicated). Control cells (U2OS) that do not express fluorescent protein are shown in the top row. **(C)** Luciferase activity assay with U2OS cells expressing αGFP-mCherry receptors co-cultured with U2OS cells expressing either GFP or GFP-ICD ligands Data points show mean values from n=6 from 3 independent experiments. Error bars represent S.E.M. ***p<0.001.

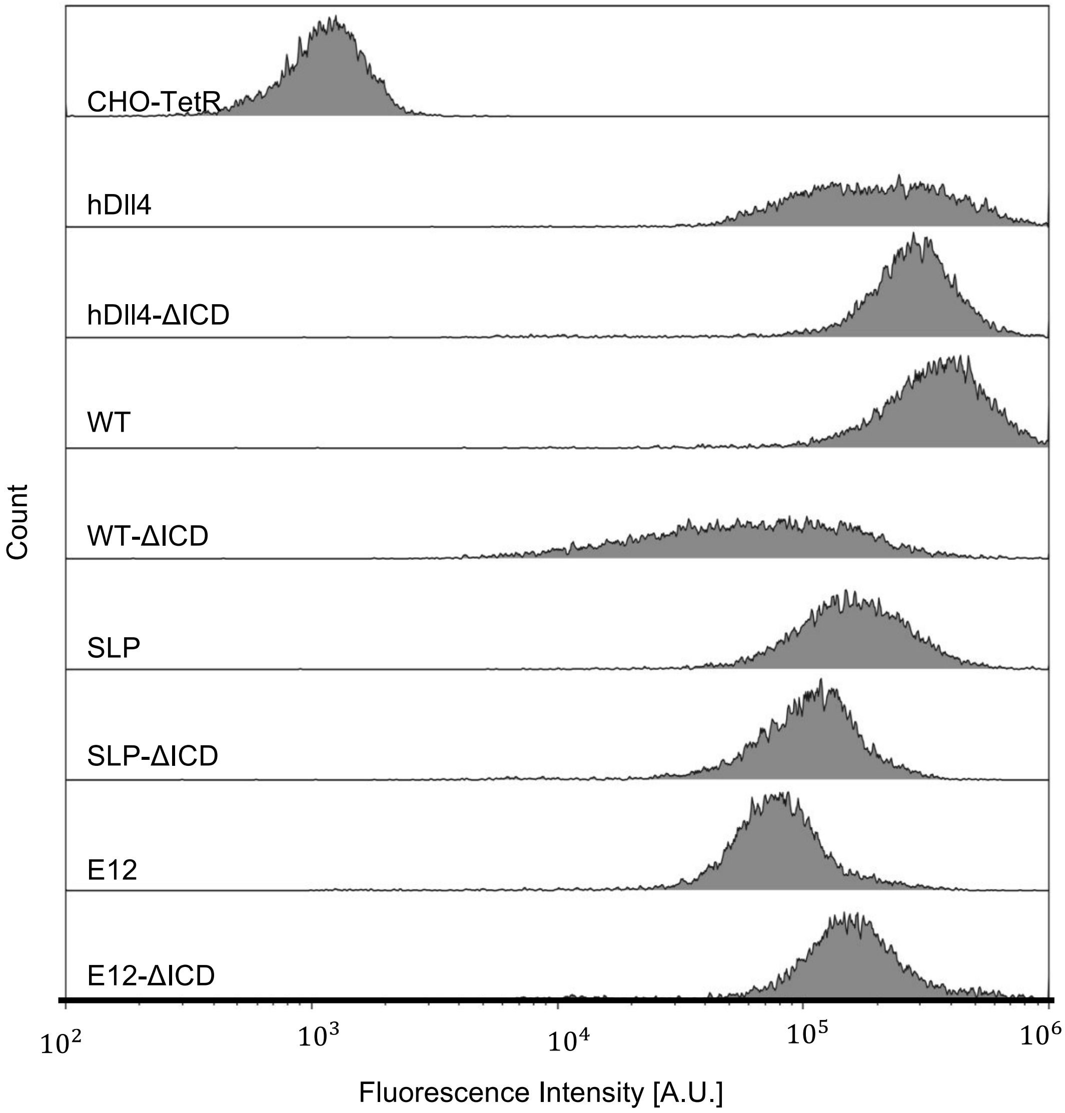


**Figure S2. FACS analysis of fluorescently tagged ligands.** Comparison of the fluorescence of CHO-TetR cells expressing different ligand variants tagged with mCherry (as indicated for each row). Control cells (CHO-TetR) that do not express fluorescent protein are shown in the top row.

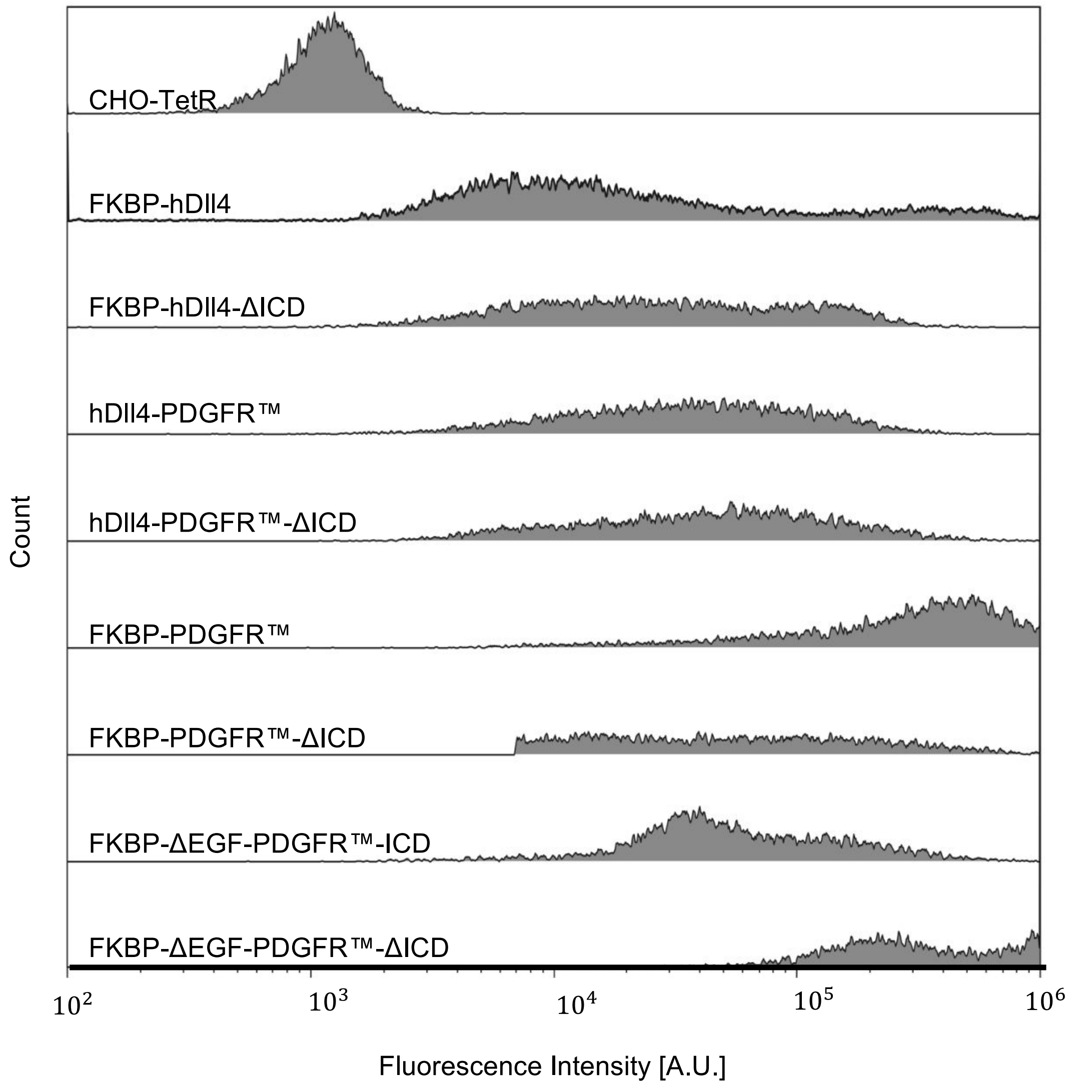


**Figure S3. FACS analysis of fluorescently tagged ligands.** Comparison of the fluorescence of CHO-TetR cells expressing different ligand variants tagged with mCherry (as indicated for each row). Control cells (CHO-TetR) that do not express fluorescent protein are shown in the top row.

## Supplementary Methods

Flow Cytometry

CHO and U2OS cells were seeded in 6-well plates at approximately 70% confluence 24 hours before FACS. Cells were treated with 100ng/ml doxycycline right after seeding. Directly prior to FACS, cells were trypsinized, spun at 1000 rpm for 5 min, and resuspended in 200 µL of FACS buffer. FACS buffer consisted of PBS with 1% FBS serum and 5mM EDTA. Flow cytometry was performed using a Cytoflex5L flow cytometer (Beckman Coulter). Kaluza software was used to analyze the data.
